# Seasonal differences in the onset of the EBV-positive and -negative forms of paediatric Hodgkin's lymphoma

**DOI:** 10.1038/sj.bjc.6601277

**Published:** 2003-09-30

**Authors:** A Reiman, J E Powell, K J Flavell, R G Grundy, J R Mann, S Parkes, D Redfern, L S Young, P G Murray

**Affiliations:** 1 Department of Pathology, Division of Cancer Studies, The Medical School, University of Birmingham, Edgbaston, Birmingham B15 2TT, UK; 2 Department of Public Health & Epidemiology, University of Birmingham, Edgbaston, Birmingham B15 2TT, UK; 3 Department of Oncology, The Children's Hospital, Steelhouse Lane, Birmingham B4 6NH, UK; 4 Department of Pathology, The Children's Hospital, Steelhouse Lane, Birmingham B4 6NH, UK; 5 Cancer Research UK Institute for Cancer Studies, University of Birmingham, Edgbaston, Birmingham B15 2TT, UK

**Keywords:** Epstein–Barr virus, paediatric Hodgkin's lymphoma, seasonality

## Abstract

In this study, we have shown that there are seasonal differences in the onset of the (Epstein–Barr virus) EBV-positive and -negative forms of paediatric Hodgkin's lymphoma (HL). This suggests aetiological differences between the two forms of this disease. EBV-positive HL might be a rare consequence of primary EBV infection.

The Epstein–Barr virus (EBV) is detectable in the malignant Hodgkin/Reed-Sternberg (HRS) cells in approximately 50% of cases of paediatric Hodgkin's lymphoma (HL) from the UK (Flavell *et al*, 2000). Although EBV is likely to play a pathogenic role in virus-associated HL cases, this has yet to be demonstrated. The striking epidemiological differences between EBV-positive and -negative paediatric HL (Weinreb *et al*, 1996; Glaser *et al*, 1997) Flavell *et al*, 2000) have been used to support the view that these two forms of HL are aetiologically distinct. A recent study by Westerbeek *et al* (1998) reported the more frequent onset of paediatric HL in the winter months, with a significant peak in December. Here we have investigated whether such seasonal effects are associated with EBV status.

## MATERIALS AND METHODS

The study population comprised all cases of histologically confirmed HL in children (<15 years) diagnosed in the West Midlands National Health Service Region, UK between 1957 and 2001. Patient data (age, date of diagnosis, sex, histological subtype, ethnic group and social class (based on father's occupation)) were extracted from medical notes by the West Midlands Regional Children's Tumour Registry (WMRCTR). Histological subtype was classified according to the Rye system (Lukes *et al*, 1966) and based upon expert histology as reported elsewhere (Parkes *et al*, 1997). Epstein–Barr virus status was determined by immunohistochemistry for LMP1 expression as previously described (Murray *et al*, 1992). Hospital notes, including general practitioner referral letters, histology reports and patient discharge summaries were examined to retrieve the date of onset of disease defined as the first appearance of symptoms. The lag time between the onset of symptoms and the date of diagnosis (defined as the date of histological diagnosis) was also recorded. A *χ*^2^ test was used to test the null hypothesis that EBV-positive and EBV-negative HL cases were equally distributed across the year. For this analysis, cases were divided into those with an onset in the summer (May–October) and winter (November–April) as described by Westerbeek *et al* (1998). Cochran's test was used to adjust for the effects of ethnic group, social class, age and histological subtype (factors associated with EBV status; Flavell *et al*, 2000)) on this analysis. Edwards' test, which looks for a one-cycle sinusoidal deviation from the null distribution (Walter, 1977; Westerbreek *et al*, 1998), was also used to identify any seasonal differences based on EBV status.

## RESULTS

There were 233 cases of HL in children recorded within the study area between 1957 and 2001. Reliable data concerning the onset of symptoms were available in 172 cases. Epstein–Barr virus status was unknown in 31 of these cases. Of the 141 tumours, 69 (48.9%) were EBV-positive. The median lag time was 12.35 weeks, although there was considerable variation in lag times; 15% of patients had a lag time up to 4 weeks duration, 35% of cases had a lag time between 1 and 3 months, and in 25% of cases the duration between onset of symptoms and diagnosis was between 4 and 6 months. The remaining 25% of patients had a lag time in excess of 6 months. Analysis of all 172 HL cases using Edward's method revealed no significant seasonal trends in the date of onset of symptoms (*P*=0.94). More (44 out of 69; 63.8%) EBV-positive patients reported first symptoms in the summer months, whereas a higher proportion of EBV-negative patients (44 out of 72; 61.1%) reported the onset of symptoms in the winter. This difference was statistically significant (*P*=0.005). A significant association between summer onset and EBV-positive tumours remained after stratification by sex (*P*=0.008), ethnic group (white *vs* non-white, *P*=0.003), social class (manual, nonmanual, unknown *P*=0.004), age (0–9 years, 10–14 years, *P*=0.005) and histological subtype (lymphocyte predominant, nodular sclerosing, mixed cellularity, other/unknown, *P*=0.011).

**Figure 1:**
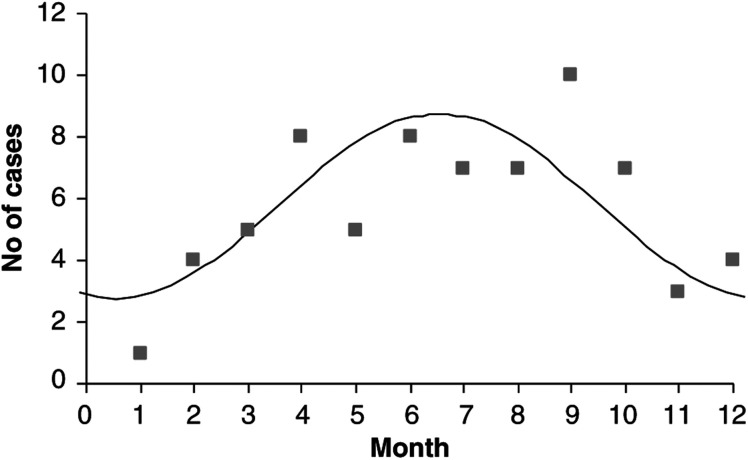
Peak in the onset of EBV-positive paediatric Hodgkin's lymphoma. Edwards' method revealed a highly significant peak onset of symptoms for EBV-positive patients occurring at the end of July/early August (*θ*=31+180=211°) with a trough at the beginning of February (*θ*=31°) (*P*=0.0093).

Analysis of EBV-positive and -negative subgroups by Edwards' method revealed a highly significant peak onset of symptoms for EBV-positive patients occurring at the end of July/early August (*θ*=31°+180°=211°) with a trough at the beginning of February (*θ*=31°) (*P*=0.0093; Figure 1

). The amplitude of the peak was 52%. Edward's test failed to identify any significant seasonal variation in the EBV-negative subgroup (*P*=0.13). No seasonal trends were observed based on the date of diagnosis, nor when the patients were subdivided on the basis of age, sex or histological subtype.

## DISCUSSION

This is only the second study to investigate seasonal variation in the onset of symptoms in paediatric HL. The first such study detected a significant peak of disease onset in December, but did not stratify patients on the basis of EBV status. Using similar methodology and patient numbers, we failed to detect any significant seasonal variations in the onset of symptoms for our group of paediatric HL patients as a whole. However, when EBV-positive and -negative patients were examined separately, a significant peak in the onset of symptoms during July/August was observed for the EBV-positive subgroup.

A number of infective and noninfective diseases exhibit seasonal variation and these seasonal rhythms can be attributed to aetiological precipitating factors. In the case of EBV-positive paediatric HL, this aetiological event might be primary EBV infection. Thus, the circannual rhythm in paediatric HL presentation could be due to a peak in the incidence of primary EBV infection occurring some months prior to the development of the tumour with the incubation period from infection to tumour development being relatively constant. Our data are consistent with the possibility that EBV-positive HL is the rare consequence of exposure to this viral agent as has been previously suggested (Parkes *et al*, 1994). Although Edwards' test failed to detect any significant seasonal variation in the EBV-negative subgroup, these patients were significantly more likely to experience the onset of symptoms in the winter months. This might support the view that EBV-negative HL is associated with as an yet unidentified infectious agent (Armstrong *et al*, 1998).

The relative proportion of EBV-positive and -negative HL patients in a population group might determine whether any seasonal effects are identifiable when these patients are combined in the analysis. Westerbeek *et al* (1998) might have detected a peak of HL onset in the winter months because of greater numbers of EBV-negative patients. Unfortunately, EBV status was not recorded in their study and we are unable to confirm this possibility.

The majority of studies to investigate seasonality in HL to date have utilised the date of diagnosis as a measure of disease onset (Newell *et al*, 1985; Douglas *et al*, 1996, 1998). However, the highly variable time between the onset of symptoms and the date of diagnosis (lag time), which was confirmed in our analysis, means that the use of date of diagnosis as a measure of disease onset could be less reliable. However, we acknowledge that problems in recording or recall could contribute to inaccuracies in studies that employ date of onset of symptoms as a measure of disease onset.

In summary, our results demonstrate a significant peak in the onset of symptoms for EBV-positive paediatric HL during the summer months and provide evidence in support of the view that EBV-positive HL might be a rare consequence of primary EBV infection. Our results also provide further evidence for aetiological differences between the EBV-positive and -negative forms of paediatric HL.
